# MAPs: a database of modular antibody parts for predicting tertiary structures and designing affinity matured antibodies

**DOI:** 10.1186/1471-2105-14-168

**Published:** 2013-05-30

**Authors:** Robert J Pantazes, Costas D Maranas

**Affiliations:** 1 Chemical Engineering Department, Penn State University, University Park, PA 16802, USA

**Keywords:** Antibody structure prediction, *De novo* protein design, V-(D)-J recombination, IMGT®

## Abstract

**Background:**

The *de novo* design of a novel protein with a particular function remains a formidable challenge with only isolated and hard-to-repeat successes to date. Due to their many structurally conserved features, antibodies are a family of proteins amenable to predictable rational design. Design algorithms must consider the structural diversity of possible naturally occurring antibodies. The human immune system samples this design space (2 10^12^) by randomly combining variable, diversity, and joining genes in a process known as V-(D)-J recombination.

**Description:**

By analyzing structural features found in affinity matured antibodies, a database of Modular Antibody Parts (MAPs) analogous to the variable, diversity, and joining genes has been constructed for the prediction of antibody tertiary structures. The database contains 929 parts constructed from an analysis of 1168 human, humanized, chimeric, and mouse antibody structures and encompasses all currently observed structural diversity of antibodies.

**Conclusions:**

The generation of 260 antibody structures shows that the MAPs database can be used to reliably predict antibody tertiary structures with an average all-atom RMSD of 1.9 Å. Using the broadly neutralizing anti-influenza antibody CH65 and anti-HIV antibody 4E10 as examples, promising starting antibodies for affinity maturation are identified and amino acid changes are traced as antibody affinity maturation occurs.

## Background

Proteins have significant value to society in diverse areas, such as chemical synthesis (e.g. catalysis), materials (e.g. silk), and medicines (e.g. antibodies). This typically requires either the successive modification of a naturally occurring protein or the *de novo* design of a new one. There have been a number of recent successes in computational *de novo* protein engineering [1-4], but the probability of success is low and lessons learned are difficult to adapt to other projects. Identifying a single successful design still requires experimentally examining tens of computationally promising proteins [5] (for a full review of the current state of the art in *de novo* protein design, please see [6]). While the *de novo* design of arbitrary proteins remains intractable, antibodies inherently have a number of modular features that make them promising systems for learning how to reliably *de novo* design proteins.

Antibodies have been extensively studied and many experimental methods are available for their construction, including hybridoma technology [7], phage display [8], yeast surface display [9], and synthetic libraries [10] (see [11] for a review). Immunoinformatics tools have been developed to identify the genes used to create antibodies from nucleotide sequences [12-17], amino acid sequences [17-22], and three-dimensional structures [19,23]. Computations have previously been used to predict antibody structures [24-26], design improvements in their interactions with antigens [27-29], and reduce their immunogenicity [30] (see [31] for a review). However, these computational techniques have primarily focused on understanding or improving *existing* antibody structures instead of the *de novo* design of new ones. The OptCDR method [32] addresses the *de novo* design of the antigen binding regions, known as complementarity determining regions (CDR), of an antibody to bind any specified epitope of an antigen. However, CDR only capture part of the binding capacity of an antibody and are not constrained to fully human designs. In this paper, we address one of the key challenges associated with the design of not just the CDR, but fully human, complete antibody variable domains: predicting initial antibody structures from a structurally diverse but computationally tractable database.

Computational antibody design must be able to consider the naturally present structural diversity spanning hundreds of millions (~3 10^8^) of potential antibodies. The human immune system achieves this diversity through V-(D)-J recombination, a process where random variable (V), diversity (D), and joining (J) germline genes are combined to create an antibody variable domain [33]. Junctional diversity introduced during V-(D)-J recombination and somatic hypermutations considerably increase the diversity of antibody variable domains, up to a theoretical limit of 2 10^12^ (a number that is not reached due to antibodies that are out-of-frame, not expressed, etc.) [34]. Thus, by *combinatorially* shuffling a number of *modular* parts, and adding somatic hypermutations, the immune system can produce billions of unique antibodies using only a few hundreds of genes interchangeably.

**Figure 1:**
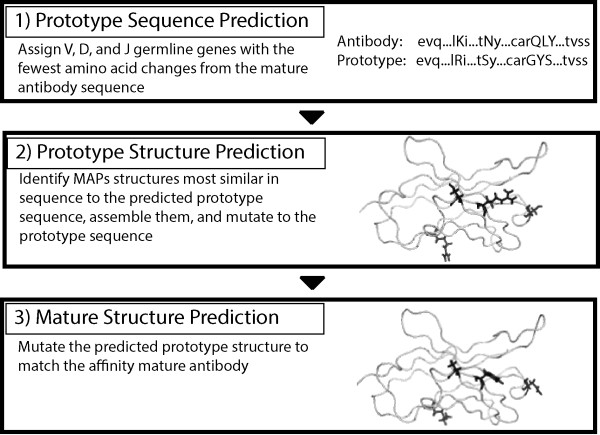
**MAPs antibody structure prediction workflow.** The MAPs database can be used to predict an antibody structure in three steps. First, a prototype sequence is predicted. In this work, that is accomplished by assigning germline genes that minimize the number of AA changes between the prototype and target antibody. However, prototype sequences from alternative methods that a user has confidence in can be used. In the second step, a prototype model is created by assembling and mutating the most similar models in the MAPs database. Finally, a predicted model of the antibody is created by mutating the prototype structure. The variable domain depicted for steps two and three is the VH of PDB 3ncJ [49] with four of the mutations highlighted.

Inspired by this paradigm, in this paper we describe the development of a database of human germline Modular Antibody Parts (MAPs) for predicting antibody tertiary structures. Figure 1 illustrates the MAPs workflow, which allows for predicting the structure of any mutated (usually affinity matured) antibody. First, a prototype sequence for the heavy (H) and light (L) chain variable domains is predicted from germline genes. Next, a model structure of the prototype sequence is created by identifying and assembling the closest MAPs structures. As detailed below, the MAPs database has structures for V* (V region Framework Region (FR) 1 to FR3), CDR3, and J* (J region FR4). Finally, the antibody structure is predicted by incorporating the amino acid (AA) changes of the antibody compared to the prototype.

The efficacy of the MAPs database to accurately predict antibody structures is assessed by predicting the structures of 260 antibodies not used in generating the database. We found that the experimentally resolved structures were predicted with an average all-atom root mean squared deviation (RMSD) of 1.900 ± 0.325 Å. Subsequently, we analyzed the broadly neutralizing anti-influenza antibody CH65 and the anti-HIV antibody 4E10 to provide starting points for the design of affinity matured antibody libraries and examine the frequency and effect of AA changes accumulated during the affinity maturation process.

## Construction and content

**Figure 2:**
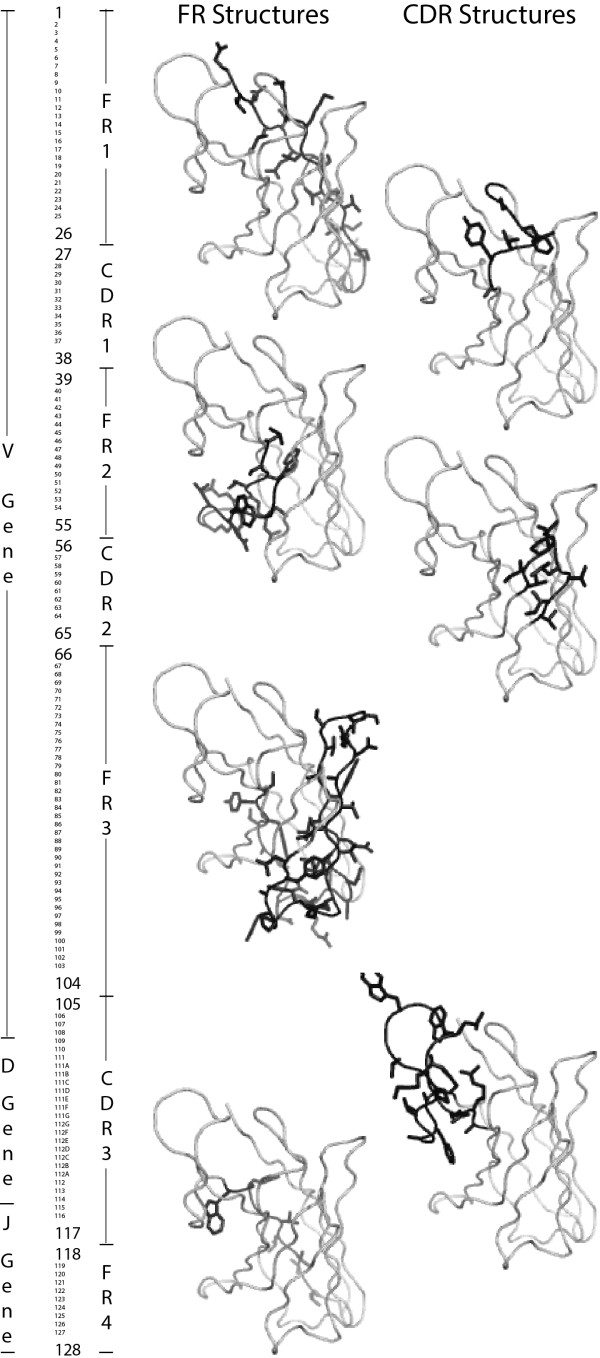
**Details of antibody sequence and structure.** The IMGT® unique numbering scheme is shown in a vertical orientation. This includes the start and end points of the FR and CDR, the corresponding structures in the VH of the broadly neutralizing anti-HIV antibody 4E10, and the approximate start and end points of the V, D, and J regions. The listed starting and ending positions of the V, D, and J regions are only approximate due to the small variations in the lengths of these genes and/or owing to trimming by exonuclease. The starting and ending positions of the FR and CDR are shown with a larger font size. VL lack D genes but generally have longer V genes to compensate.

An antibody’s antigen recognition site is formed by two “variable” domains, one each from a H and a L chain. Each domain contains three CDR (6 total) attached to a structurally conserved FR. For five out of six CDR (except for CDR3 in the H variable domain (VH)) there are a limited number of conformations that their backbones may assume, known as canonical structures [35]. These structurally conserved features allow for standardized numbering schemes describing each position in an antibody. In this paper, we use the IMGT® unique numbering [36-39] from IMGT®, the international ImMunoGeneTics information system® http://www.imgt.org[35,40]. IMGT® is a well-curated source of antibody sequences, genes, structures, and standards used extensively in this paper. Each VH is constructed by combining randomly selected V, D, and J genes from the IGH locus [34]. Light variable (VL) domains do not have D regions and are constructed by rearranging V and J genes from the IGK and IGL loci [34], forming either κ (V-KAPPA) or λ (V-LAMBDA) antibodies, respectively. Figure 2 shows the IMGT® unique numbering [36-39], the approximate start and end points of the V, D, and J regions (which depend on the length of the germline regions and trimming during V-(D)-J rearrangement), the conserved start and end points of the FR and CDR, and the location of FR and CDR within an antibody variable domain.

The first step in Figure 1 is the assignment of germline V, D, and J genes to an antibody to identify a prototype sequence. From the IMGT/GENE-DB [41] we downloaded all human germline V, D, and J genes and retained all complete (i.e., no missing nucleotides) and unique genes and alleles, spanning a total diversity of approximately 10^8^ possible antibodies (see Table 1). The adopted hypothesis for assigning germline genes to an antibody variable domain is that the gene combination with the fewest AA changes from the corresponding portion of the antibody’s sequence is the one most likely to have been used in V-(D)-J recombination.

**Table 1:** Human germline V, D, and J genes and alleles

	**IGH**		**IGK**		**IGL**	
	**Total**	**Retained**	**Total**	**Retained**	**Total**	**Retained**
**V**	324	236	97	82	86	75
**D**	44	37	0	0	0	0
**J**	13	11	9	9	10	8
**Antibodies**	1.85 10^5^	9.61 10^4^	873	738	860	600
**Total Antibodies**		**3.21 10**^**8**^		**Retained Antibodies**		**1.29 10**^**8**^

Germline genes and alleles downloaded from the IMGT/GENE-DB [41]. Only the genes and alleles that had no missing nucleotides were retained for use in assigning germline genes to an antibody.

Compared to the germline genes, the junctions (in IMGT® positions 104–118) which result from the V-(D)-J rearrangement usually show nucleotide (nt) deletions at the 3’ V region and 5’ J region, and for a VH on both ends of the D region, due to exonuclease trimming. The junctions also show random nt insertions by the terminal deoxynucleotidyl transferase [34]. For the VH, this makes the assignment of the D gene only possible at the nt level [16,17]. Therefore, the determination of the number of AA changes between a set of V, D, and J regions and a VH or VL is carried out as follows:

1. The total number of AA changes in the V and J regions outside of CDR3 is counted. Note that AA insertions and deletions are rare (observed rates of 0.00-0.09% per region) outside of CDR3 and are penalized as AA changes.

2. The CDR3 nucleotides of the 3’ V and 5’ J regions are fixed in place at the start and end, respectively, of CDR3.

3. In CDR3, the V and J combination is first evaluated for the presence of nt gaps or overlaps (i.e., insertions or deletions). If there is a gap between the end of the V region and the start of the J region, the gap is filled so that the minimum number of AA changes is introduced. Conversely, if the regions overlap, deletions that cause the fewest AA changes are chosen. Then, the total number of AA changes in CDR3 is counted. Note that nt insertions and deletions are penalized based on the number of AA they affect (e.g. 1–3 insertions affect 1 AA, 4–6 affects 2, etc.). This procedure is sufficient for VL and for the few VH that lack D genes.

4. For VH, the D region is positioned at every possible position from the beginning of CDR3 to the end. For each possible position, the number of AA changes is assessed as in Step 3.

The V, D, and J gene combination that results in the fewest AA changes is selected as the prototype sequence for the target antibody. In the event that two or more sets of genes have the same number of AA changes, the minimum number of nt substitutions is used as a tie-breaker (e.g. mutating Ile (att) to Val (gtt) or Thr (act) requires a minimum of one nt substitution, but mutating Val to Thr requires at least two). This procedure is very fast for VL (< 0.5 s) and reasonably quick for VH (5 – 60 s) due to the added complexity of considering the D regions.

**Figure 3:**
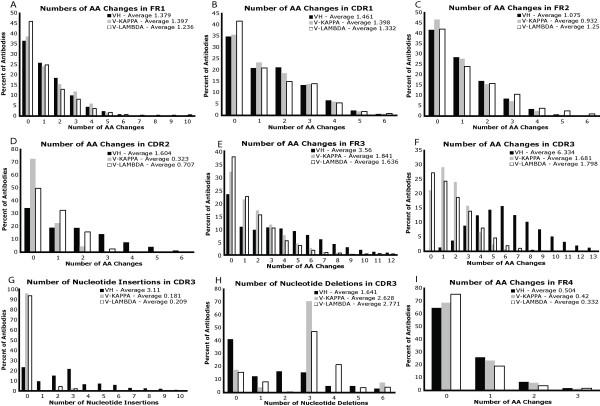
**Observed AA change numbers in human antibodies.** Here we show the numbers of AA changes in each of the seven regions of antibody structure for VH, V-KAPPA, and V-LAMBDA. Panels **G** and **H** show the number of nucleotide insertions and deletions in CDR3, as this is the only region where those events are common.

**Figure 4:**
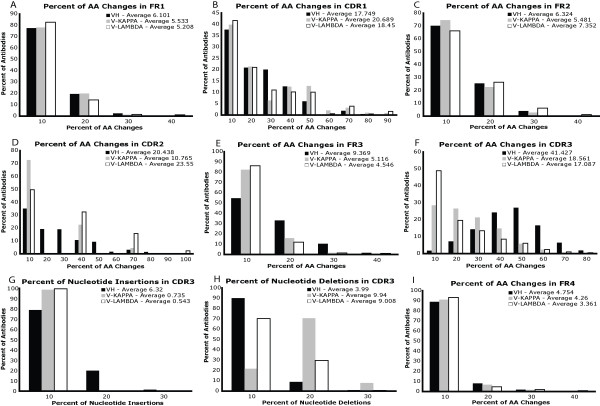
**Observed AA change frequencies in human antibodies.** Here we show the percent of AA that are changed in each of the seven regions of antibody structure for VH, V-KAPPA, and V-LAMBDA. Panels **G** and **H** show the percentage of nt insertions and deletions in CDR3, as this is the only region where those events are common.

To test the gene assignment protocol, we downloaded 7,652 VH, 2,247 V-KAPPA and 1,605 V-LAMBDA unique, human antibodies from IMGT/LIGM-DB [42] and assigned germline genes to them. The observed rates and number of AA changes in each of the FR and CDR along with AA insertions and deletions in CDR3 are shown in Figures 3 and 4. The observed frequencies and locations of AA changes match expected trends. The average number of AA changes (i.e., VH 17.6, V-KAPPA and V-LAMBDA both 9.0) compare quite well with the expected 10–15 AA changes per variable domain [33]. In addition, the average rate of AA changes in the “hypervariable” CDR is much higher than in the FR. This is especially pronounced in the VH CDR3, which is well-known to often have the most antigen contacting residues. Interestingly, FR3 also accumulates a much higher number of AA changes in the VH compared with the VL. The confirmation of expected experimental trends in the average frequency and number of AA changes alludes to the efficacy of the gene assignment protocol.

With this established gene assignment protocol, we next turn our attention to determining if antibodies with the same germline genes assume the same structures. Reliable prediction of antibody tertiary structures, modeled from their prototype AA sequences, hinges upon the hypothesis that antibody regions that share the same prototype sequence assume similar structures. We downloaded 1,168 human, humanized, chimeric, and mouse antibody structures from the IMGT/3Dstructure-DB [19,23] as well as all complete mouse IGH, IGK, and IGL V, D, and J genes from the IMGT/GENE-DB [41]. Prototype sequences were identified for all antibodies, with human genes used for human antibodies and mouse genes used for mouse, chimeric, and humanized antibodies. Note that chimeric antibodies have mouse variable domains and humanized antibodies have mouse CDR attached to human FR. Mouse genes were used for the humanized antibodies so that CDR3 were modeled using the appropriate genes.

With the prototype sequences determined for the antibody structures, a clustering procedure similar to one used in a previous work for just the CDR [32] was used to determine if identical regions give rise to similar structures. At the end of the clustering procedure, the structure with the smallest average backbone atom (N, Cα, C) RMSD with all other structures in a cluster was selected as the model structure. The clustering was carried out so that all members of a cluster have a backbone atom RMSD of no more than 2.0 Å with the model structure. This distance cutoff was also used to assess previous antibody structure prediction methods [24-26].

**Figure 5:**
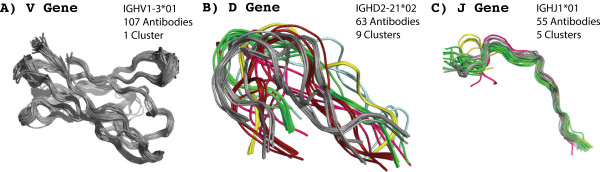
**Clustering of V, D, and J region structures.** These panels show representative clusters of structures with the same V (Panel **A**), D (Panel **B**), and J (Panel **C**) regions, respectively. All structures in the same cluster have the same color. For the VH V region and VL, the clustering worked well. However, for the VH D and J regions the clustering procedure did not work (i.e., antibodies with the same germline regions have different structures). Interestingly, all of the deviations occur within CDR3. This observation led to the development of an alternative delimitation of structure: V* (everything before CDR3, FR1-FR3, IMGT positions 1–104), CDR3, and J* (everything after CDR3, FR4, IMGT positions 118–128).

An initial analysis was conducted to determine if using models of the V, D, and J genes was an effective approach to generate the MAPs database. Figure 5 gives representative results of the clustering process. For the V regions and the light J regions the procedure led to almost entirely unique inferences for structure, but for the VH D and J regions antibodies with identical regions often had different structures. This implies that *assignment of the germline regions may be sufficient to predict the structures of VL but is insufficient for VH*. However, we observed that for both VH and VL the clustering procedure appeared to work well outside of CDR3 and poorly within CDR3. This includes the 3’ V regions where “fraying” of the clusters was observed in the last few residues. This suggests that a modified description, listed in Table 2, utilizing CDR3 as a structural component instead of D regions may improve the description of antibody structures. We therefore selected an alternative delimitation of structures for both VH and VL: (i) V* (FR1-FR3, IMGT® positions 1–104), containing all the sequence before CDR3 (i.e., most of the V region), (ii) CDR3 (IMGT® positions 105–117), and (iii) J* (FR4, IMGT® positions 118–128), encompassing everything after CDR3 (i.e., most of the J region). This alternative concatenation of the genetic information provides a more succinct description of structural diversity.

**Table 2:** Alternative delimitation of structure

	**IMGT start**	**IMGT end**
V* (FR1-FR3)	1	104
**CDR3**	105	117
J* (FR4)	118	128

Positions are according to the IMGT® unique numbering. Positions 104 (2nd-CYS) and 118 (J-PHE or J-TRP) belong to FR3 and FR4, respectively, and are the anchors of CDR3.

When the clustering procedure is applied to the antibodies based on the prototype V*, CDR3, and J* sequences the clustering results show one structure per one prototype sequence. After discounting obvious explanations for structural differences (e.g. unusual antibody structures like light chain dimers or triabodies, mutations known to alter crystal packing, structural perturbations caused by the linkers in single-chain variable fragments, antigen binding causing conformational changes, etc.), only 9 VH V*, 18 VH CDR3, and 6 V-KAPPA CDR3 structures out of the 1168 antibodies used were found to differ from other structures with the same prototype sequence. All other regions had at most a single outlier. Most of the differing CDR3 structures were those that were naturally diverse, owing to the expected high diversity of their junctions, suggesting that larger structural changes are to be expected.

The observation that each prototype V*, CDR3, and J* sequence has a single structure indicates that a database of these structures can be used to model antibody variable domains. The MAPs database was constructed to contain as much structural diversity as possible. For each V*, CDR3, and J* prototype sequence, the antibody from the 1168 structures that required the fewest AA changes was selected as the model. In the event that two or more antibodies shared the same minimum number of AA changes, the structure with the smallest average backbone atom RMSD with the other possible models was chosen. The model structures had their sequences mutated to the germline sequence using a previously published optimal rotamer selection procedure [43,44] and any structural inconsistencies were then corrected with a CHARMM energy minimization [45]. All CHARMM energy minimizations in this publication were molecular mechanics minimizations and were carried out using the “all27_prot_na” topology and parameter files, the angl, bond, dihe, elec, impr, urey, and vdw energy terms, and no solvation. The created models were stored in the MAPs database as PDB files using the IMGT® unique numbering for the V domain [36-39].

Each unique human J* prototype in Table 1 was modeled using this procedure. All J* models required two or fewer AA changes. Human V* prototypes shown in Table 1 were also similarly modeled using a cutoff on the maximum number of AA changes allowed. No V* prototype was selected if it required more than one standard deviation greater than the average number of AA changes expected (cutoffs of 16 AA changes for IGHV and 14 for IGKV and IGLV, as determined from the data in Figure 3). This cutoff was imposed to ensure that the V* models were accurate structural representations of their sequences.

Each CDR3 prototype was modeled using a cutoff on the maximum number of allowable AA changes. An analysis of the RMSDs between all pairs of VH CDR3 with the same number of AA shows that on average one extra AA change gives rise to a 1/3 Å increase in backbone RMSD (R^2^ = 0.97). Therefore a cutoff of six AA changes was used, as this number of changes would likely cause an average change of 2.0 Å in backbone RMSD, which is the similarity cutoff used during the clustering procedure and in previous antibody structure prediction methods [24-26]. Prototype CDR3 whose model required six or fewer AA changes were changed to the predicted sequence in the same manner as the V* and J* models, while those requiring seven or more changes were included using the experimentally determined model structure. The modeling of the CDR3 ensured that the maximum amount of structural diversity was included in the MAPs database for this essential binding feature, at the expense of introducing some mouse and non-prototype sequences.

The size statistics of the MAPs database are presented in Table 3. The MAPs database is composed of 929 “parts” that can be assembled to create 2.3 10^10^ unique antibodies. This is in fact more antibodies than can be assembled by the human immune system through rearrangement of the V, D, and J genes. However, the complex mechanisms and junctional diversity of V-(D)-J recombination significantly increases the number of antibodies that the immune system can potentially generate. The MAPs database contains all currently observed structural diversity of antibodies with CDR that can encompass a wide range of possible positions.

**Table 3:** Number of model structures in the MAPs database

	**VH**	**V-KAPPA**	**V-LAMBDA**
V*	141	67	38
**CDR3**	428	199	39
J*	5	5	7
**Total**	3.0 10^5^	6.7 10^4^	1.0 10^4^
**Possible Antibodies**		2.3 10^10^	

The number of models for each region of structure for each type of antibody variable domain is tabulated.

The MAPs database can be used to model antibody structures as shown in Figure 1. For a target affinity matured antibody with an unknown structure, a prototype sequence is first computationally identified. Next, the V*, CDR3, and J* structures in the MAPs database that have the closest sequence to the prototype are identified. The models are mutated to the prototype sequence using the optimal rotamer selection protocol based on the Iterative Protein Redesign & Optimization (IPRO) method [43,44] and relaxed using a CHARMM energy minimization [45] step. Finally, mutating the prototype antibody followed by another CHARMM energy minimization generates the predicted structure of the target antibody.

## Utility and discussion

The efficacy of the MAPs database for predicting antibody tertiary structures from their AA sequences was assessed. A cross-validation set of 260 antibodies from the 1168 downloaded was selected. These 260 antibodies were not used in creating any of the model structures of the database, contained both VH and VL, and had experimental resolutions no worse than 2.5 Å. The structures were predicted using the workflow from Figure 1 with a mean RMSD of 1.900 ± 0.325 Å accounting for all atoms in all residues in both variable domains. The mean experimental resolution of the structures was 2.074 ± 0.274 Å. Additional file 1 lists the all-atom RMSDs of each of the 260 predicted antibody models. As the V* and J* model structures are based on human genes, it is to be expected that the predicted human antibodies (56 out of 260) had a slightly better mean RMSD than the predicted mouse, chimeric, and humanized antibodies (i.e. 1.771 ± 0.184 Å versus 1.933 ± 0.345 Å). In contrast, there is no significant difference between the mean RMSDs of the 207 bound antibody complexes (i.e. 1.876 ± 0.320 Å) and the 53 unbound complexes (i.e. 1.992 ± 0.330 Å).

We relied on three popular online servers for predicting antibody structures (i.e., Web Antibody Modeling (WAM) [26], Prediction of ImmunGlobulin Structure (PIGS) [24], and RosettaAntibody [25]) to benchmark the effectiveness of the introduced method. WAM’s published results show that 16 out of 19 (i.e., 84%) predicted antibodies had VH CDR3 backbone RMSD values no worse than 2.0 Å. Our results meet the same RMSD criterion for a much larger set (i.e., 249 out of 260) and percentage (i.e., 96%) of predicted structures. PIGS provided the backbone RMSD of all AA in four antibodies (i.e., 1.08, 1.11, 1.16, and 1.42 Å). The corresponding mean and median using the MAPs database are comparable (1.134 ± 0.365 Å and 1.022 Å, respectively). RosettaAntibody published RMSD results for the backbone atoms of all AA in the CDR of 54 antibodies that have a median RMSD of 1.4 Å with 80% of them less than 2.0 Å. MAPs based structure prediction yields a median RMSD of 1.256 Å with 209 out of 260 (80%) better than 2.0 Å. These results demonstrate that using the MAPs database to predict antibody structures is at least as accurate as existing methods and in most cases better.

**Figure 6:**
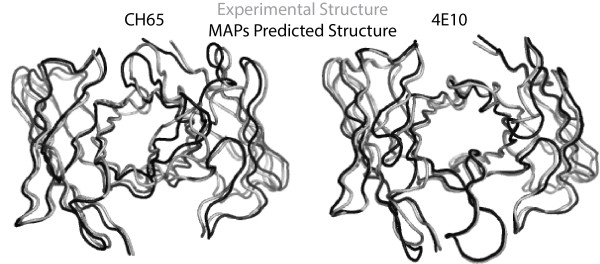
**The experimental and MAPs predicted structures of antibodies CH65 and 4E10.** The experimental structures are shown in light gray and the MAPs predicted structures are shown in black. CH65 has an all-atom RMSD of 2.046 Å and 4E10 has an all-atom RMSD of 2.099 Å.

We also briefly explored the efficacy of using the MAPs database to support antibody engineering and design using two broadly neutralizing antibodies: the anti-influenza antibody CH65 (PDB: 3sm5) [46] and the anti-HIV antibody 4E10 (PDB: 2fx7) [47]. The experimental and MAPs predicted structures for CH65 and 4E10 (see Figure 6) have an all atom RMSD of 2.046 and 2.099 Å, respectively. Due to its recent publication date, the structure of CH65 was not one of the 1168 considered when creating the MAPs database. The overall quality of its MAPS based predicted structure is comparable to that of 4E10 with some structure prediction discrepancies only within the VH CDR3. This CDR is 20 AA long and has at least a ten AA difference from any VH CDR3 structure in the MAPs database. Nevertheless, the backbone atom RMSD of the VH CDR3 from the experimental structure is only 2.160 Å.

The first step in the proposed workflow for antibody structure prediction in Figure 1 is the prediction of the best prototype sequences for any given antibody. Table 4 lists the AA changes in the ten best prototype sequences for antibody CH65, containing 21 positions in VH and 12 positions in V-LAMBDA. The VH AA changes are predominantly allotted within FR3 and CDR3 while the V-LAMBDA AA changes are distributed throughout the sequence. A more uniform distribution of AA changes in VH could be obtained, at the expense of introducing more total AA changes, by limiting how often each gene is used in the predicted results. For example, this may be desirable in the construction of a combinatorial library to bind a particular antigen epitope.

**Table 4:** AA changes in the ten best predicted prototypes for the broadly-neutralizing anti-influenza antibody CH65

	**Position**	**Antibody**	**P1**	**P2**	**P3**	**P4**	**P5**	**P6**	**P7**	**P8**	**P9**	**P10**
**VH**	1	E	Q	Q	Q	Q	Q	Q	Q	Q	Q	Q
	36	D	G	G	G	G	G	G	G	G	G	G
	38	H	Y	Y	Y	Y	Y	Y	Y	Y	Y	Y
	39	I	M	M	M	M	M	M	M	M	M	M
	40	N	H	H	H	H	H	H	H	H	H	H
	57	H	N	N	N	N	N	N	N	N	N	N
	64	D	G	G	G	G	G	G	G	G	G	G
	83	A	S	S	S	S	S	S	S	S	S	S
	91	V	L	L	L	L	L	L	L	L	L	L
	92	N	S	S	S	S	S	S	S	S	S	S
	93	G	R	R	R	R	R	R	R	R	R	R
	95	K	R	R	R	R	R	R	R	R	R	R
	107	G	V	G	G	G	G	G	G	G	G	G
	108	G	Q	S	I	W	G	G	G	G	G	Y
	109	L	L	I	V	I	I	I	I	I	I	S
	110	E	E	A	G	Q	A	T	T	T	T	S
	111	P	R	A	A	L	A	G	G	G	G	S
	111A	R	R	R	T	W	A	T	T	T	T	W
	111B	S	S	P	T	L	T	T	T	T	T	Y
	111C	V	D	D	D	H	D	D	D	D	D	D
	112C	D	Y	Y	Y	Y	Y	Y	Y	Y	Y	Y
**V-LAMBDA**	2	S	Y	Y	Y	Y	Y	Y	Y	Y	Y	Y
	17	Q	Q	K	K	Q	Q	K	K	Q	K	K
	27	D	N	N	N	N	N	N	N	N	N	N
	36	R	S	S	S	S	S	S	S	S	S	S
	42	N	Y	Y	Y	Y	Y	Y	Y	Y	Y	Y
	54	V	V	I	V	V	V	I	V	V	I	V
	55	C	Y	Y	Y	Y	Y	Y	Y	Y	Y	Y
	56	Y	D	Y	D	D	D	Y	D	D	Y	D
	116	V	V	V	V	W	A	W	W	A	A	A
	117	I	V	V	V	V	V	V	V	V	V	V
	123	K	K	K	K	K	Q	K	K	Q	Q	Q
	126	V	V	V	V	V	V	V	V	A	V	V

The AA changes of the top ten prototype (P) sequences (21 VH, 12 V-LAMBDA positions) are listed. In VH all positions outside of CDR3 have the same AA listed because the same V gene was selected. Within CDR3 different D gene selections give rise to different AA changes. In V-LAMBDA different V and J gene selections give rise to different AA changes throughout the entire domain. Sequence numbering is according to the IMGT® unique numbering [36-39].

The identification of the prototype sequence and the generation of prototype and affinity matured models simplifies the assessment of interesting AA changes relevant to a particular antibody. The anti-HIV antibody 4E10 contains 28 VH and 14 V-KAPPA AA changes from the prototype sequence. Table 5 reports the changes in the computed interaction energy between the antibody and antigen for each one of the 42 AA changes accumulated during affinity maturation. A detailed description of these calculations is available in Additional file 2. The computed interaction energies should be considered for the *qualitative* trends they imply rather than their absolute values. Only two of the observed AA changes are computationally predicted to be detrimental to binding while 37 are predicted to be weakly and three strongly beneficial. This suggests that the AA changes needed to convert the prototype structure to the affinity matured antibody generally improve binding. This lends credibility to the computational identification of prototypes because affinity maturation, as expected, led to binding improving whereas random mutations would have been detrimental to binding.

**Table 5:** Predicted changes in interaction energy for AA changes in the broadly-neutralizing anti-HIV antibody 4E10

**Domain**	**Mutation**	**ΔΔG (kcal/mol)**
VH	K 14 R	−2.54
VH	K 20 T	−1.975
VH	T 29 S	−3.071
VH	S 36 T	−2.343
VH	I 39 L	−3.495
VH	Q 48 R	−2.846
VH	I 56 V	−1.889
VH	I 59 L	−3.449
VH	G 63 T	−2.573
VH	A 65 T	−1.801
VH	Q 69 P	−1.691
VH	K 70 R	−2.226
VH	V 76 I	−1.678
VH	K 82 R	−2.143
VH	M 89 L	−2.158
VH	S 92 N	−2.549
VH	S 96 P	−2.451
VH	I 109 T	−0.923
VH	I 111 G	−1.026
VH	F 111a W	−2.648
VH	V 111c W	−3.922
VH	V 112d L	−2.517
VH	I 112c G	−3.328
VH	I 112b K	−45.027
VH	D 113 G	−45.886
VH	D 116 A	5.989
VH	V 117 H	−0.445
VH	M 123 L	−1.908
V-KAPPA	L 11 Q	−2.11
V-KAPPA	S 30 G	−3.713
V-KAPPA	S 36 N	0.412
V-KAPPA	S 37 N	−2.128
V-KAPPA	Y 38 K	−55.314
V-KAPPA	K 45 R	−2.614
V-KAPPA	A 68 P	−2.604
V-KAPPA	T 69 S	−2.681
V-KAPPA	I 71 V	−2.177
V-KAPPA	P 72 A	−2.41
V-KAPPA	S 109 Q	−2.969
V-KAPPA	P 115 L	−4.398
V-KAPPA	W 116 S	−5.325
V-KAPPA	I 126 V	−2.531

Note that 37 of 42 AA changes are predicted to be weakly beneficial, 3 are predicted to be strongly beneficial, and 2 are predicted to be weakly detrimental to antigen binding. Sequence numbering is according to the IMGT® unique numbering [36-39].

## Conclusions

This paper introduced a modular database of antibody parts that can be used in the *de novo* design of antibodies in an analogous fashion to V-(D)-J recombination. Using the structural diversity encompassed within 1168 experimental antibody structures we compiled the MAPs database that contains 929 parts that can be combined to create 2.3 10^10^ unique antibodies. The prediction of 260 antibody structures not used in making any of the MAPs database models revealed that this database can be used to reliably predict antibody tertiary structures. In contrast to previous antibody structure prediction methods [24-26], MAPs allows for antibody structure prediction without the need for *de novo* folding calculations every time. The all-atom, modular nature of the MAPs database allows for the pre-calculation of pairwise structural component interaction energies. The computational savings do not come at the expense of accuracy of prediction as the RMSD of the predicted structures is at least as accurate as earlier methods.

Antibody structures are known to be affected to some extent by the relative orientation of the VH and VL, the specific canonical structures used, and the FR to which they attach. However, despite ignoring all these factors the procedure described in Figure 1 operating on the MAPs database provides very high fidelity of antibody structure prediction (see also Additional file 1), alluding to a high degree of modularity of available conformations.

The broadly-neutralizing anti-HIV antibody 4E10 was used to demonstrate the ability of the MAPs database to model the nature of AA changes upon affinity maturation. Almost all of the accumulated AA changes were predicted to be beneficial to binding, thus providing indirect evidence regarding the validity of the prototypes. The proposed workflow is generally agnostic to the method used to identify prototypes for an antibody. If a user has higher confidence in a prototype sequence different from the one identified by the method described here, it is possible to directly import it in the calculations.

Currently, almost all antibodies are designed entirely using experimental methods. Once a promising antibody is identified additional affinity improvements are sought after using random mutagenesis and directed evolution protocols. Knowing where to target mutations and what type of mutations to explore can greatly improve the efficiency of experimental methods. In the germline repertoire of V, D, and J genes, evolution has retained many similar genes with AA changes at key positions that are likely to influence binding to a range of substrates. As described for the broadly neutralizing anti-influenza antibody CH65, it is possible to rapidly identify multiple potential prototypes for any given antibody. By contrasting the mutations between the target antibody and the prototypes both promising positions and AA combinations likely to confer improved binding affinity can be quickly compiled providing cues for combinatorial library design.

## Availability and requirements

The MAPs database has been incorporated into the IPRO suite of programs [32,43,44,48], available on our website (http://maranas.che.psu.edu). It is only freely-available to academic users and all others should contact the corresponding author for more information.

## Abbreviations

AA: Amino acid; CDR: Complementarity determining region; D: Diversity; FR: Framework region; IMGT®: the international ImMunoGeneTics information system®; J: Joining; MAPs: Modular Antibody Parts; nt: Nucleotide; PDB: Protein Data Bank; PIGS: Prediction of ImmunoGlobulin Structures; RMSD: Root mean squared deviation; V: Variable; WAM: Web Antibody Modeling.

## Competing interests

The authors have no competing interests to declare.

## Authors’ contributions

RP carried out all of the computational work, including the development, modeling, and validation of the MAPs database, as well drafted the manuscript. CM conceived of the study, supervised all work, and assisted in drafting the manuscript. All authors read and approved the final manuscript.

## Supplementary Material

Additional file 1 The all-atom RMSD results for each of the 260 antibody structures used in the cross-validation of the MAPs database. Click here for file

Additional file 2 **Description of the calculation of the interaction energies reported in Table** 5**for the broadly-neutralizing anti-HIV antibody 4E10.** Click here for file
