# Supplementary material for: MAPs: a database of modular antibody parts for predicting tertiary structures and designing affinity matured antibodies
Source: BMC Bioinformatics. 2013 May 30;14:168. doi: 10.1186/1471-2105-14-168 (PMC3687570; doi:10.1186/1471-2105-14-168)
Supplement: Additional file 2 — Description of the calculation of the interaction energies reported in Table 5for the broadly-neutralizing anti-HIV antibody 4E10. [file 1471-2105-14-168-S2.docx]

**Description of the calculation of the interaction energies reported in Table 5 for the broadly-neutralizing anti-HIV antibody 4E10**

First, a model of unbound 4E10 was generated using the procedure shown in Figure 1. An experimentally determined complex for this antibody is available (PDB: 2fx7 and IMGT/3Dstructure-DB, http://www.imgt.org). We aligned our predicted 4E10 VH and V-KAPPA domains with those from the experimental structure in a single step (i.e. the domains were aligned together, *not individually*). This provided an initial placement of the antigen relative to the variable domains.

We have previously developed and published the Iterative Protein Redesign & Optimization (IPRO) [[1](#_ENREF_1), [2](#_ENREF_2)] procedure to redesign proteins for improved binding to ligands. The procedure involves iteratively perturbing the protein (antibody) backbone, selecting an optimal positioning of amino acid rotamers, and a local docking procedure to improve binding. We ran 500 iterations of IPRO without allowing any mutations to generate the initial affinity matured complex.

To calculate the interaction energies, we created generated each AA change individually from the affinity matured complex. 20 iterations of IPRO were run for each mutant to ensure that any local backbone changes or antigen repositionings would be correctly modeled. The CHARMM portions of IPRO were the same as described in the main paper: they used the “all27_prot_na” topology and parameter files, the angl, bond, dihe, elec, impr, urey, and vdw energy terms, and no solvation. The changes in interaction energy for each AA change was then calculated and is given in Table 5, where interaction energy is the minimized energy of the antibody-antigen complex minus the energy of the antibody minus the energy of the antigen.

1. Fazelinia H, Cirino PC, Maranas CD: **Extending Iterative Protein Redesign and Optimization (IPRO) in protein library design for ligand specificity**. *Biophys J* 2007, **92**(6):2120-2130.

2. Saraf MC, Moore GL, Goodey NM, Cao VY, Benkovic SJ, Maranas CD: **IPRO: an iterative computational protein library redesign and optimization procedure.** *Biophys J* 2006, **90**(11):4167-4180.
